# Self-Assembly Assisted Fabrication of Dextran-Based Nanohydrogels with Reduction-Cleavable Junctions for Applications as Efficient Drug Delivery Systems

**DOI:** 10.1038/srep40011

**Published:** 2017-01-10

**Authors:** Hao Wang, Tingting Dai, Shuyan Zhou, Xiaoxiao Huang, Songying Li, Kang Sun, Guangdong Zhou, Hongjing Dou

**Affiliations:** 1 The State Key Laboratory of Metal Matrix Composites, School of Materials Science and Engineering, Shanghai Jiao Tong University, Shanghai 200240 (P. R. China); 2 Department of Plastic and Reconstructive Surgery, Shanghai ninth People’s Hospital, Shanghai Jiao Tong University School of Medicine, Shanghai Key Laboratory of Tissue Engineering, National Tissue Engineering Center of China, Shanghai 200011 (P. R. China)

## Abstract

In order to overcome the key challenge in improving both fabrication efficiency and their drug delivery capability of anti-cancer drug delivery systems (ACDDS), here polyacrylic acid (PAA) grafted dextran (Dex) nanohydrogels (NGs) with covalent crosslinked structure bearing redox sensitive disulfide crosslinking junctions (Dex-SS-PAA) were synthesized efficiently through a one-step self-assembly assisted methodology (SAA). The Dex-SS-PAA were subsequently conjugated with doxorubicin through an acid-labile hydrazone bond (Dex-SS-PAA-DOX). The *in vitro* drug release behavior, anti-cancer effects *in vivo*, and biosafety of the as-prepared acid- and redox-dual responsive biodegradable NGs were systematically investigated. The results revealed that the Dex-SS-PAA-DOX exhibited pH- and redox-controlled drug release, greatly reduced the toxicity of free DOX, while exhibiting a strong ability to inhibit the growth of MDA-MB-231 tumors. Our study demonstrated that the Dex-SS-PAA-DOX NGs are very promising candidates as ACDDS for anti-cancer therapeutics.

During recent decades, chemotherapy has been one of the most common methods to treat cancer^1^. However, anti-cancer drugs usually have adverse side-effects on normal tissues, which greatly limits the efficacy of this treatment. The use of liposomes^2^, micelles^3^, and nanoparticles^4^,^5^,^6^ as anti-cancer drug delivery vehicles have attracted tremendous attention because they can passively target tumor tissues through the enhanced permeability retention (EPR)^7^ effect and significantly reduce the side-effects of the drug. In comparison with other drug delivery carriers, nanohydrogels (NGs) are more effective and safer as ideal drug delivery systems due to their functions as nanoparticle and unique characteristics, such as their high stability *in vivo* resulting from their crosslinked structures, excellent drug loading capacities, high hydrophilicity and biocompatibility^8^,^9^,^10^,^11^. These unique properties provide NGs with prolonged systemic circulation times, stability during prolonged circulation and minimize premature drug release to normal tissues^11^.

Controlled release and targeted drug delivery with high efficacy are the key issues in anti-cancer drug delivery systems^12^,^13^,^14^. Numerous environmentally responsive NGs have been developed for drug delivery applications^13^,^15^,^16^,^17^. Consequently, these NGs had exhibited more effective targeted delivery performance, and they thus facilitated the use of lower drug dosages. However, most of these NG systems were single responsive^18^,^19^,^20^, they still cannot meet the practical needs of controlled release of chemotherapy drugs. For example, the concentrations of GSH encountered in normal cells could also cleave disulfide bonds and result in toxic side-effects and poor drug selectivity toward tumor cells^21^. To conquer the previous limitation, multi-responsive NGs have been fabricated and caused extensive consern of researchers in recent years^11^,^15^,^16^,^17^,^22^,^23^. These new resultant NGs had exhibited efficient drug release at tumor sites and reduced the side-effects of the drug itself on normal tissue in comparison with single responsive NG systems. However, most of these multi-responsive NGs load the drugs through weak interactions such as electrostatic interaction and hydrogen bond interaction^24^,^25^,^26^, and consequent instability results in serious drug leakage in the circulation of the blood. What’s more, these NGs are always synthesized by non-cleavable crosslinkers, leading to poor biodegradability^25^,^27^. Therefore, there is an urgent need to design a stable, biodegradable multi-responsive NGs drug delivery systems with controlled drug release.

Recently, our group developed a facile and highly efficient self-assembly assisted (SAA) approach to synthesize polysaccharide-based nanogels for cell nanoprobes and glucose sensors^28^,^29^,^30^. This method also provides a new strategy to construct stimuli-responsive drug delivery NGs. As reported herein, we utilize the fact that tumor exhibit weak acid^31^,^32^ and reductive microenvironment^33^,^34^ and synthesized highly efficient a pH- and redox-dual responsive dextran-based NGs for drug delivery. The dextran was chosen as the main building block of the NGs due to their biodegradability, biocompatibility, and crucial role in improving the endocytosis behavior^10^,^35^. In addition, the easliy cleavable redox-labile disulfide bond and the acid-labile hydrazone bond were introduced to obtain biodegradable dual responsive NGs. Furthermore, the animal experiment of NGs systems was conducted to verify their safety and effectiveness *in vivo*. Results indicated that the dual-responsive NGs exhibited accelerated drug release behavior in acidic and reducing environments as well as enhanced drug controlled release efficiency at tumor sites and reduced the drug’s toxicity.

In this work, dextran-based NGs are fabricated via a straightforward SAA approach in which the disulfide junction bearing crosslinker was chosen to lock the structure of the NGs (Fig. 1 and Supplementary Fig. S1). Thereafter, the dextran-based NGs were loaded with a model drug (doxorubicin, denoted as DOX) through the acid-labile hydrazone bond, which further offered the redox-responsive NGs with pH-responsive capability. The *in vitro* drug release behavior, *in vivo* anti-cancer effects and biosafety of the dual responsive DOX-loaded NGs were systematically investigated on the basis of their efficient fabrication.

## Results and discussion

### Fabrication of the NGs

Dex-SS-PAA NGs were fabricated with a modified (SAA) method developed by our group^28^,^29^,^30^. During the SAA fabrication of Dex-SS-PAA NGs, the initiator ceric ammonium nitrate (CAN) was added into the dextran solution to induce the formation of free radicals on the dextran backbone. Most of these free radicals would occupy the C2 or C3 positions of the glucose unit^36^,^37^. Subsequently, the hydrophilic monomer acrylic acid (AA) was added. As the polymerization of the AA from the dextran backbone progressed, nanoaggregates were formed due to the hydrogen bonding interactions between the hydroxyl groups of dextran and the carboxyl groups of the PAA chains. Furthermore, the nanoscale structure of the aggregates was fixed through the covalent bonds due to the involvement of the DADS crosslinker.

**Figure 2:**
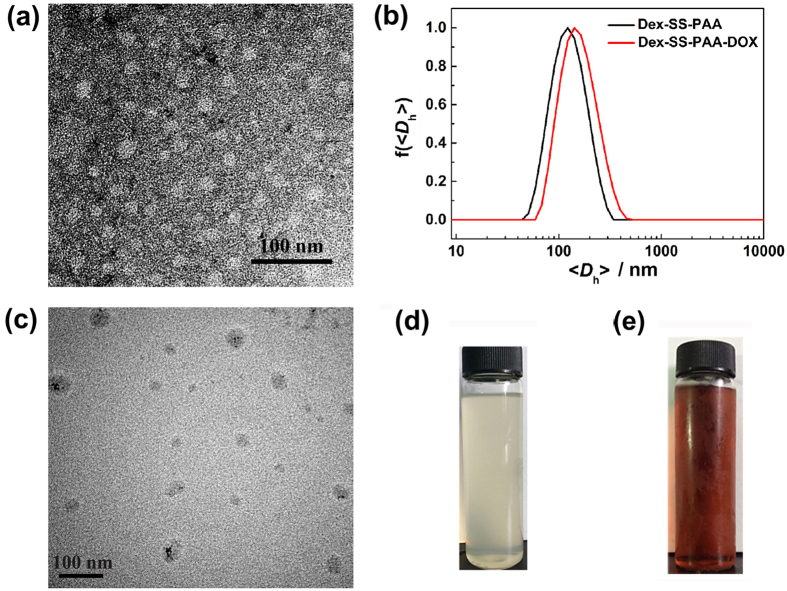
Fabrication of the Dex-SS-PAA and Dex-SS-PAA-DOX NGs. (**a**) TEM image of Dex-SS-PAA NGs (negatively stained by an aqueous 2 wt.% phosphotungstic acid solution). (**b**) The size distribution of Dex-SS-PAA NGs and Dex-SS-PAA-DOX NGs. (**c**) TEM image of Dex-SS-PAA-DOX NGs. Images of (**d**) the Dex-SS-PAA NGs (35 g/L) and (**e**) the Dex-SS-PAA-DOX NGs (2 g/L).

The successful preparation of Dex-SS-PAA NGs was verified by ^1^H NMR spectroscopy, (Supplementary Fig. S2). The ^1^H NMR spectrum of Dex-SS-PAA NGs exhibited proton signals corresponding to the dextran moiety in the range of 3.0–5.0 ppm, and also displayed new signals between 1.0–2.5 ppm, which could be attributed to the protons of the PAA chains. It is noteworthy that this process yielded Dex-SS-PAA NGs at a high efficiency and the final concentration of the Dex-SS-PAA NGs solution could reach ~35 g/L, as shown in the inset of Fig. 2d.

The size of nanoparticle plays an important role in determining its tumor treatment behavior. According to our previous work^28^, the diameters of NGs synthesized by the SAA approach can be easily tuned by adjusting the fabrication conditions, especially the monomer-to-dextran unit molar ratio. In this work, therefore, when the molar ratio of AA to dextran units was varied from 0.5, to 1 and to 2, our study disclosed that the resultant Dex-SS-PAA NGs had hydrodynamic diameters of 37.5, 98.0, and 944.3 nm, respectively (Supplementary Table S1). In consideration of the tumor vascular permeability and the EPR effect on the circulation within the body^7^, the Dex-SS-PAA NG with a hydrodynamic diameter of ~98 nm was chosen for further DOX conjugation and anti-tumor investigations. As shown in Fig. 2a,b, the Dex-SS-PAA NG exhibited a spherical shape with a diameter of ~40 nm in the dry state under TEM observation and an average hydrodynamic diameter (<*D*_h_>) of 98.0 nm in aqueous solution. The disparity of the two results lies in the shrinkage of the NGs during the TEM sample preparation^38^. The Dex-SS-PAA NGs exhibited a relatively narrow distribution with a particle dispersion index (PDI) of 0.191 and a negative surface charge with a zeta potential of −10.0 mV.

In addition, the flexibility of the SAA fabrication methodology was shown to be effective in the fabrication of dextran-based NGs when another carboxyl group-bearing monomer, methacrylic acid (MAA), was used as the monomer instead of the current acrylic acid. As shown in Supplementary Fig. S3, Dex-SS-PMAA NGs (at a MAA-to-dextran unit molar ratio of 1:1) with an average hydrodynamic diameter (<*D*_h_>) of 90.6 nm were successfully fabricated, and the concentration of the NGs in the resultant reaction solution reached as high as 29 g/L.

### Redox-responsiveness of the Dex-SS-PAA NGs

**Figure 3:**
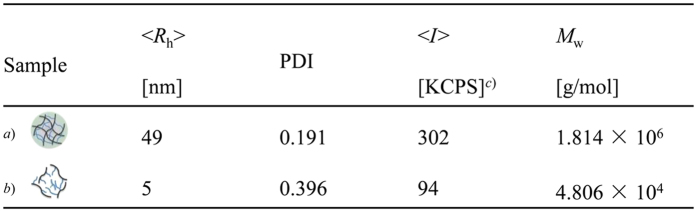
DLS and SLS characterization of Dex-SS-PAA NGs before and after GSH addition. (**a**) Before GSH addition; (**b**) After GSH addition; (**c**) Kilo counts per second.

To evaluate the redox-responsiveness of the Dex-SS-PAA NGs, the reducing agent L-glutathione reduced GSH (10 mM) was added into an aqueous Dex-SS-PAA NG solution to simulate the tumor microenvironment^16^,^39^. The molecular weight of the system was calculated *via* the Zimm plot method based on SLS measurements^40^. DLS results showed a significant decrease of <*D*_h_> (from 98 to 10 nm) during the first 3 h after GSH addition, as shown in Fig. 3, as well as a decrease in the light scattering intensity in the solution as shown in both Fig. 3 and Supplementary Fig. S4a. TEM image of Dex-SS-PAA at 30 min after GSH addition showed some fragments, which suggested that NG has begun to degrade (Supplementary Fig. S4b). In addition, SLS results showed a molecular weight (*M*_w_) decrease from 10^6^ g/mol in magnitude to 10^4^ g/mol in magnitude after GSH addition. It is worth noting that the *M*_w_ observed after GSH addition was at the same level with that of dextran (4 × 10^4^ g/mol), indicating that the disassembly of Dex-SS-PAA NGs had occurred via cleavage at the disulfide crosslinking junctions. In comparison with the constant size and light scattering intensity of the control sample that was observed in the absence of GSH, the disassembly of Dex-SS-PAA NGs were confirmed due to the cleavage of the disulfide junctions in the presence of GSH, thus clearly demonstrating the sensitivity of Dex-SS-PAA NGs to reducing environments. Based on the design of the NGs, this property will facilitate the drug release upon exposure to the reducing environments encountered at tumor sites.

### Fabrication of DOX-loaded NGs

In order to conjugate DOX with the NGs and endow the resultant drug-loaded carrier with sensitivity to the acidic environments encountered at tumor sites and thus enable targeted drug delivery, the acid-labile hydrazone bond was chosen as the linkage between DOX and the NGs. The successful fabrication of Dex-SS-PAA-DOX NGs was verified *via* TEM and ^1^H NMR characterization. As shown in Fig. 2c, TEM showed that the DOX-loaded NGs also were nearly spherical in shape with a diameter of ~40 nm in the dry state. In addition, they had a <*D*_h_> of *~*110 nm (Fig. 2b), which is slightly larger than that of the NGs observed prior to DOX-loading due to the hydrophobicity of the DOX moiety. An image of the Dex-SS- PAA-DOX NGs (2 g/L) is shown in Fig. 2e. In addition, the ^1^H NMR spectrum of the Dex-SS-PAA-DOX NGs shown in Supplementary Fig. S5 revealed several new signals at chemical shifts of 6–8 ppm. These signals correspond to the protons belonging to the phenyl rings of DOX, while some new signals at 1–2 ppm can be attributed to the methylene groups of DOX.

As a redox-insensitive control, crosslinker DADS was replaced by MBA and Dex-MBA-PAA-DOX NGs were successfully fabricated in a similar manner. The TEM image, size distribution and ^1^H NMR spectrum of the Dex-MBA-PAA-DOX NGs are shown in Supplementary Fig. S6 and Supplementary Table S2.

### *In vitro* release of DOX

The drug loading content (LC) and drug encapsulation efficiency (EE) are the important factors that determine the performance of anti-cancer drug delivery systems, and these parameters were measured *via* the UV-Vis absorbance method. According to the absorbance calibration curve of free DOX (Supplementary Fig. S7), the LC and EE values of both Dex-SS-PAA-DOX and Dex-MBA-PAA-DOX NGs were calculated. Dex-SS-PAA-DOX NGs showed a LC of 5.5 wt.% and an EE of 33.3%. Meanwhile, its equivalent counterparts Dex-MBA-PAA-DOX NGs showed a comparable LC of 5.7 wt.% and an EE of 34.4%. Therefore, these two kinds of materials exhibited comparable drug loading behavior.

**Figure 4:**
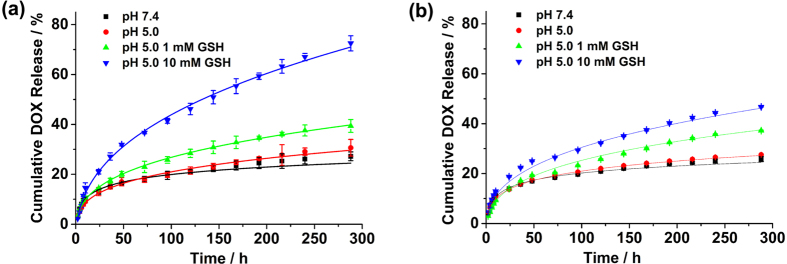
*In vitro* drug release behaviors of Dex-SS-PAA-DOX NGs (**a**) and Dex-MBA-PAA-DOX NGs (**b**) under various release conditions.

The *in vitro* drug release profiles of both DOX-loaded NGs exhibited rather prolonged DOX release profiles and the redox responsiveness was somewhat weak at pH 5.0 (Fig. 4 and Supplementary Fig. S8). This might have been because DOX was protected by the crosslinked shell. Moreover, it should be noted that when 10 mM GSH was added, DOX was released much more rapidly from the Dex-SS-PAA NGs at pH = 5.0, the cumulative DOX release could reach ~73% after 300 h of release. In contrast, the cumulative DOX release of the redox-insensitive control could reach ~47%. Furthermore, ca. 80% of DOX was released from NGs at pH 3 in 120 hours, and by adding 10 mM GSH into the release medium, almost 100% of DOX was released during the same term. The slight increase of DOX release observed in acidic and reducing environments in comparison with in acidic environments could be due to the cleavage of the acid-labile hydrazone bonds resulting from the decrease of the pH encountered upon the addition of GSH^41^. Besides, the drug release at pH 7.4 might be attributed to the small amount of physically trapped DOX inside the Dex-SS-PAA NGs. Here pH was measured using pH meter: pH = 5.0 in acidic environments, pH = 3.8 in acidic and reducing environments. In order to clarify the effect of GSH addition on the pH values of the PBS buffer used in our study, we measured the pH values of the resultant buffer after adding GSH at different concentrations (Supplementary Table S3). On the basis of our study, the buffer (0.01 M) we chose in drug release studies display a significant pH decrease upon GSH addition, whereas the other type of buffer in which 0.05 M of phosphate salt were used is more tolerant to GSH addition, and can keep almost their initial pH values after adding 10 mM GSH. Our studies disclosed the dependence of the buffering capacity on the concentrations of phosphate salts.

### *In vitro* cytotoxicity assay

**Figure 5:**
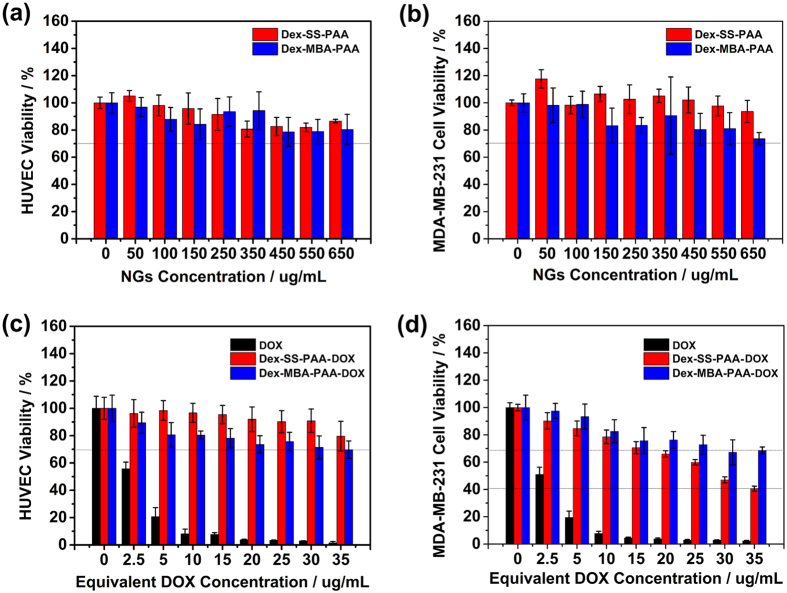
(**a**) HUVEC viability and (**b**) MDA-MB-231 cell viability of non-drug-loaded Dex-SS-PAA NGs and Dex-MBA-PAA NGs. (**c**) HUVEC inhibition abilities and (**d**) MDA-MB-231 cell inhibition performance of free DOX, Dex-SS-PAA-DOX NGs and Dex-MBA-PAA-DOX NGs.

To serve as a safe drug carrier system, it is desirable that the anti-cancer drug delivery systems display good biocompatibility toward normal cells, while inhibiting the growth of cancer cells. To this end, the *in vitro* cytotoxicity of the NGs toward normal cells (HUVEC) and cancer cells were evaluated *via* the MTT assay method. Considering that the incidence of breast cancer has become a leading cause of death for women^42^, we chose two kinds of human breast cancer cell lines (i.e., MCF-7 and MDA-MB-231 cells) as model cell lines. Firstly, we investigated the biocompatibility of both the blank NGs and the DOX-loaded NGs. Both kinds of blank NGs showed low toxicity toward HUVEC and MDA-MB-231 cells (Fig. 5a,b). In addition, the DOX-loaded NGs showed little toxicity toward HUVEC even at a carrier concent ration of 650 μg/mL after 72 h of co-incubation (Fig. 5c). This may be attributed to their environmentally sensitive DOX release capabilities that allow them to selectively target tumor sites.

The inhibition capabilities of DOX-loaded NGs were subsequently measured together with free DOX (Fig. 5d), and the results suggested that free DOX showed severe toxicity toward MDA-MB-231 cells even at a very low concentration of 5 μg/mL when the cell viability rate was *~*18%. Although the inhibition performance of DOX-loaded NGs was somehow compromised, they still retained most of their anti-cancer effect at higher equivalent DOX concentrations. The Dex-SS-PAA-DOX NGs showed stronger inhibition performance in comparison with Dex-MBA-PAA-DOX NGs, and this difference in performance might be attributed to the enhanced DOX targeting release capability of the Dex-SS-PAA-DOX NGs in acidic and reducing environments. Besides, as being indicated by Fig. 5(d), the difference in cytotoxicity between Dex-SS-PAA-DOX and Dex-MBA-PAA-DOX NGs is dose dependent. This dose difference can be attributed to the difference of the two NGs in drug release behavior. As we can see from the Fig. 4, the drug release percentage of the Dex-SS-PAA-DOX is much higher than that of Dex-MBA-PAA-DOX NGs at given GSH concentration. Therefore, the difference between the absolute amount of the DOX released from the two NGs is dose dependent. Correspondingly, the difference between Dex-MBA-PAA-DOX and Dex-SS-PAA-DOX in cytotoxicity is reasonable if considering the relationship between DOX dose and their cytotoxicity.

In addition, DOX-loaded NGs showed high inhibition performance toward MCF-7 cells (Supplementary Fig. S9). In comparison with Dex-MBA-PAA-DOX NGs, Dex-SS-PAA-DOX NGs bearing disulfide crosslinking junctions display higher inhibition performance toward MCF-7 cells, which exhibited the same trend as that of MDA-MB-231 cells. The *in vitro* cytotoxicity assay suggested that the pH/redox dual-responsive NGs have promising potential as anti-cancer drug carriers for targeting breast cancer cells.

### Cellular uptake

**Figure 6:**
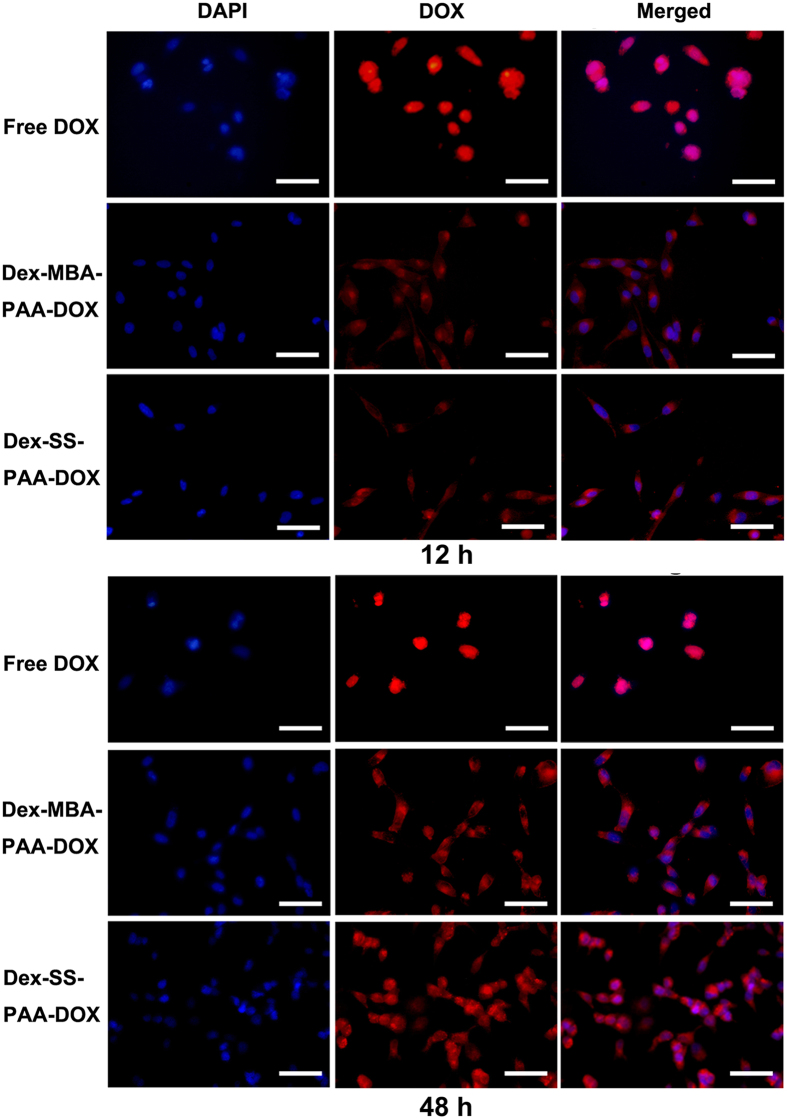
Fluorescence microscope images of MDA-MB-231 cells after co-incubation with free DOX, Dex-MBA-PAA-DOX NGs and Dex-SS-PAA-DOX NGs for 12 or 48 h at an equivalent DOX concentration of 15 μg/mL, the red colour corresponds to the fluorescence of DOX, the blue colour corresponds to the fluorescence of DAPI (a cell nucleus dye). Scale bars: 100 μm.

MDA-MB-231 cells were used to investigate the cellular uptake and intracellular drug release behaviors of DOX-loaded NGs and free DOX. The pictures of MDA-MB-231 cells that were co-incubated with free DOX or both DOX-loaded NGs for 12 or 48 h at an equivalent DOX concentration of 15 μg/mL were recorded under a fluorescent microscope. Cellular nuclei were stained with DAPI to determine whether DOX entered the nuclei. As demonstrated in Fig. 6, the fluorescence of DOX was detected in both the cells treated with the free drug and with the DOX-loaded NGs after 12 h of incubation, suggesting that the drug had already accumulated inside the cells within 12 h of incubation. However, they exhibited quite different drug intracellular distributions and fluorescence intensities. Free DOX molecules can rapidly penetrate the nuclei of MDA-MB-231 cells and then interact with the DNA structure^43^, and the fluorescence intensity of DOX inside the cells was the highest in this case. The DOX fluorescence within the cells treated with either Dex-MBA-PAA-DOX NGs or with Dex-SS-PAA-DOX NGs was weaker than that exhibited by the free DOX sample, and filled the entire cell after 12 h of incubation, indicating that the internalization of NGs was occurring and that only some DOX was released from the NGs and transported into the nuclei. This indicated that both of the DOX-loaded carriers entered the MDA-MB-231 cells in a much slower manner, most probably through the endocytosis pathway^12^,^44^ as the size of NGs was ~100 nm.

However, increase in fluorescence intensity of both Dex-MBA-PAA-DOX NGs and Dex-SS-PAA-DOX NGs was observed in the nuclei after 48 h of incubation, which was apparently due to the fact that more DOX was released from the DOX-loaded NGs and subsequently entered the nuclei during this time.

Fluorescence microscope images of MCF-7 cells recorded after incubation with free DOX and DOX-loaded NGs for 48 h are shown in Supplementary Fig. S9c. Similarly, the Dex-SS-PAA-DOX NGs display an enhanced capability for both becoming internalized by these two cell lines and for releasing DOX within the cancer cells.

### *In vivo* antitumor effect

**Figure 7:**
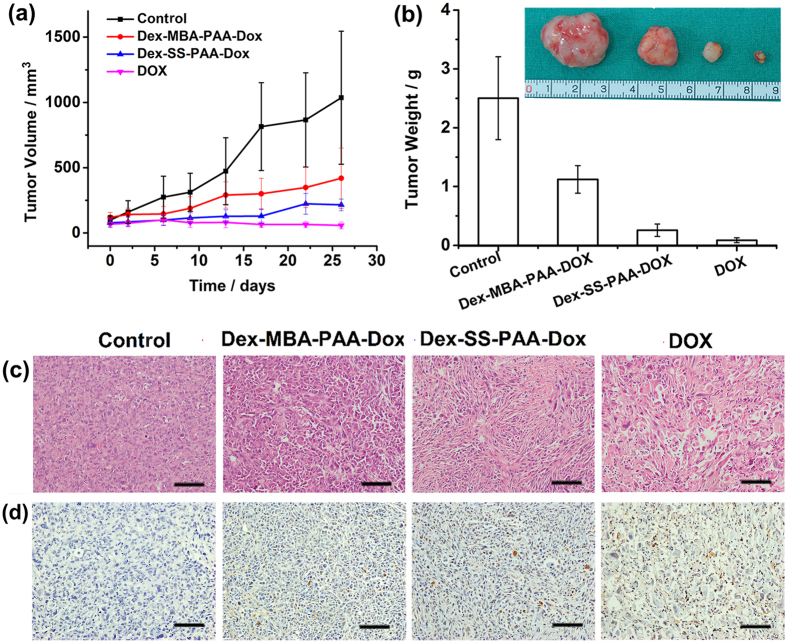
*In vivo* anticancer effect of female nude mice. (**a**) Tumor volume changes and (**b**) Tumor weight of solid tumors from the mice after 26 days of treatment. The inset images show photos of the respective tumors after their removal. (**c**) H&E stained MDA-MB-231 tumor sections of solid tumors from the mice after 26 days of treatment. Nuclei were stained blue while extracellular matrix and cytoplasm were stained red. (**d**) TUNEL stained MDA-MB-231 tumor sections of solid tumors from the mice after 26 days of treatment. Scale bars: 100 μm.

The *In vivo* antitumor effect is a crucial standard to evaluate the performance of an anti-cancer drug delivery system. Therefore, we evaluated the *in vivo* antitumor effect of Dex-SS-PAA-DOX NGs on nude mice bearing a subcutaneous MDA-MB-231 tumor xenograft (Fig. 7). A plot of the tumor volume versus time is shown in Fig. 7a. The tumor volume of the control group rapidly increased, reaching 1032 mm^3^ after 26 days. In contrast, drug-loaded and free DOX exhibited higher tumor inhibition capabilities after two treatments. On day 26, the tumor volumes of samples treated with Dex-MBA-PAA-DOX NGs, Dex-SS-PAA-DOX NGs, and free DOX groups were 422, 210, and 58 mm^3^, which were 40.9%, 20.3%, and 5.6% of that observed in the control group, respectively. The same result was confirmed by observing the tumor weights after 26 days of treatment, as demonstrated by the calculated inhibition ratios shown in Fig. 7b. The most efficient inhibition of tumor growth was observed among the group treated with the free drug, and the inhibition ratio (IR) was 96.5% versus 89.7% for the loaded dual-responsive Dex-SS-PAA-DOX NGs and 55.2% for loaded pH-responsive Dex-MBA-PAA-DOX NGs. The tumor inhibition capability of the Dex-SS-PAA-DOX NGS were weaker than that of free DOX at an equivalence dosage, which might have resulted from the rather prolonged DOX release behavior as confirmed by Fig. 4. However, Dex-SS-PAA-DOX NGs had much stronger cytotoxicity against tumors than both that of the control group and the Dex-MBA-PAA-DOX NGs. There are one possible reason that may account for the higher antitumor efficiency exhibited by Dex-SS-PAA-DOX NGs if comparing it with Dex-MBA-PAA-DOX. The acidic and reductive environment encountered at the tumor sites triggers the accelerated release of DOX^45^. This feature has contributed to improve the antitumor efficiency of the Dex-SS-PAA-DOX NGs.

It is known that the anti-cancer capability of DOX is due to its interaction with the DNA structures to inhibit their transcription after entering the nuclei of cancer cells, thus inducing cellular apoptosis^43^. Therefore, the therapeutic effects of DOX-loaded NGs can be verified by the extent of apoptosis observed in treated tumor tissue, which were investigated by observing H&E stained and TUNEL stained MDA-MB-231 tumor sections of solid tumors collected from the treated mice after 26 days. As shown in Fig. 7c,d, tumor cells treated with both DOX-loaded NGs and free DOX exhibit higher degrees of cellular apoptosis in comparison with the control groups. In addition, tumor cells treated with Dex-SS-PAA-DOX NGs exhibited higher degrees of apoptosis compared with those treated with Dex-MBA-PAA-DOX NGs.

### Safety evaluation

**Figure 8:**
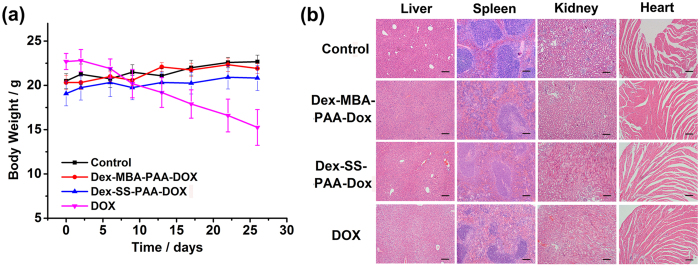
Results of *in vivo* biosafety evaluations including. (**a**) Body weight changes during the 26 days of treatment. (**b**) H&E stained liver, spleen, kidney, and heart tissue samples from the mice after 26 days of treatment. Nuclei were stained blue while extracellular matrix and cytoplasm were stained red. Scale bars: 200 μm.

Good biosafety is a key requirement for an effective drug delivery system, thus the practical *in vivo* biosafety of DOX-loaded NGs were evaluated. The body weight records of the mice shown in Fig. 8a suggested that the free DOX exhibited a severe side-effect on mice, as could be seen from the dramatic loss in body weight experienced by these mice. In comparison, the encapsulation of DOX within the anti-cancer drug delivery systems greatly diminished this adverse effect. The body weight and apparent health of the mice remained unchanged in comparison with those of the control group. The histological examination further showed that the organs (liver, kidney, spleen, and heart) of mice treated with Dex-SS-PAA-DOX NGs and Dex-MBA-PAA-DOX NGs did not exhibit obvious appreciable pathological lesions after 26 days of treatment (Fig. 8b). In contrast, hepatic injury and renal hemorrhaging were observed in tissue samples collected from the mice that had been treated with free DOX. The results demonstrated that the Dex-SS-PAA-DOX NGs exhibited good tumor inhibition performance and could suppress the side-effects of chemotherapy otherwise encountered with DOX.

## Conclusion

In summary, we have successfully fabricated redox-responsive Dex-SS-PAA NGs *via* a facile self-assembly assisted method. Our study demonstrated that this methodology provides a highly efficient pathway to fabricate dextran-based ACDDS. The hydrodynamic diameters of the resultant Dex-SS-PAA NGs can be readily tuned and they exhibit good responsiveness to reductive environments. *In vitro* DLS and SLS, TEM studies revealed that in the reductive environments simulating those typically encountered in tumor tissues, the NGs could disassemble into small fragments due to the cleavage of their disulfide crosslinking junctions. After they had been conjugated with DOX through a pH-responsive hydrazone bond, the resultant Dex-SS-PAA-DOX NGs displayed an better pH/redox dual-responsiveness in comparison with pH-single responsive NG systems, and thus an accelerated DOX release behavior in both acidic and reductive environments that are the characteristics of tumor tissues. Both cytotoxicity and *in vivo* tumor inhibition studies demonstrated that the disulfide bond bearing Dex-SS-PAA-DOX NGs exhibited stronger cytotoxicity toward MDA-MB-231, and MCF-7 cells than their non-cleavable pH-responsive Dex-MBA-PAA-DOX NG counterparts in reductive environments. Additionally, both kinds of dextran-based NGs exhibited good biosafety according to our *in vivo* study. Our investigations confirmed that the Dex-SS-PAA-DOX NGs exhibited good tumor inhibition performance while suppressing the side-effects associated with DOX.

## Methods

### Fabrication of the NGs bearing disulfide (Dex-SS-PAA NGs and Dex-SS-PMAA NGs) and carbon-carbon bond crosslinking junctions (Dex-MBA-PAA NGs)

Dex-SS-PAA NGs were fabricated according to a reported approach^28^,^29^,^30^, but with appropriate modifications. The synthetic strategy is shown in Supplementary Fig. S1. In a typical process, 2.500 g (6.26 × 10^−5^ mol) of dextran was fully dissolved in 50 mL of water and the resultant solution was kept under N_2_ protection and gently stirred for 30 min at 30 °C. At this point the initiator CAN (1.210 g or 2.21 × 10^−3^ mol pre-dissolved in 1.25 mL of 0.1 M HNO_3_) was added, and after 5 min the monomer AA (1.072 g, 1.49 × 10^−2^ mol) or MAA (1.283 g, 1.49 × 10^−2^ mol) was added into the solution. The crosslinker DADS (0.218 g, 1.49 × 10^−3^ mol dissolved in 5 mL of DMSO) or MBA (0.230 g or 1.49 × 10^−3^ mol dissolved in 10 mL deionized water) was added into the system 30 min after the addition of the monomer. The entire process was allowed to proceed for 4 h and then the solution was adjusted to pH = 7 with 1 M NaOH and dialyzed against deionized water for 3 days. In order to adjust the diameter of the NGs, three Dex-SS-PAA NGs denoted as Dex-SS-PAA (S), Dex-SS-PAA, Dex-SS-PAA (L) with various diameters were synthesized through addition of different AA moler. The DLS performance results are shown in Supplementary Table S1.

### Fabrication of Dex-SS-PAA-DOX NGs and Dex-MBA-PAA-DOX NGs

As shown in Supplementary Fig. S1, DOX was covalently conjugated to Dex-SS-PAA NGs *via* an acid-labile hydrazone bond through a two-step reaction^46^. Firstly, 2.500 g of Dex-SS-PAA NGs was thoroughly dispersed into 400 mL of water and was subsequently heated to 110 °C before an excess amount of hydrazinium hydroxide (90 mL) was added dropwise into the solution. The reaction mixture was refluxed and mechanically stirred at a speed of 700 rpm for 4 h. The entire solution was then transferred into a dialysis bag and dialyzed against pure water for three days and subsequently freeze-dried to obtain the hydrazide-modified Dex-SS-PAA-NHNH_2_ NGs. Secondly, 0.500 g of dried Dex-SS-PAA-NHNH_2_ NGs were dissolved in 100 mL of anhydrous DMSO. Then, the resultant solution was kept under N_2_ protection and gently stirred for one hour at 60 °C. Subsequently, 5 mL of a DMSO solution containing 0.100 g of DOX was added into the solution. Finally, 100 μL of triethylamine (TEA) was added into the solution. The mixture was stirred at 60 °C in the dark for 48 h under nitrogen protection. The resultant Dex-SS-PAA-DOX NGs were dialyzed against an alkaline aqueous solution for three days (Alkaline aqueous solution was changed 4–5 times every day) and then lyophilized. In order to demonstrate the advantage of dual-responsive Dex-SS-PAA-DOX NGs, single stimulus-responsive Dex-MBA-PAA NGs bearing C-C crosslinking junctions were conjugated with DOX *via* the hydrazone bonds through the same procedure to obtain the pH-responsive.

In order to determine the amount of the DOX that was physically trapped inside the Dex-SS-PAA-DOX, the Dex-SS-PAA was mixed directly with DOX without its transformation into Dex-SS-PAA-NHNH_2_, the resultant product was analyzed to confirm the DOX content inside the NGs. The detailed procedure is as follows: 0.500 g of dried Dex-SS-PAA NGs were dissolved in 100 mL of anhydrous DMSO. Subsequently, 5 mL of a DMSO solution containing 0.100 g of DOX and 100 μL of triethylamine (TEA) was added into the solution. The mixture was stirred at 60 °C in the dark for 48 h under nitrogen protection. The resultant Dex-SS-PAA (DOX) NGs were dialyzed against an alkaline aqueous solution for three days and then lyophilized. By using the UV-Vis absorption spectra, the drug loading content of this sample was calculated as *ca.* 1.8 wt.%, which revealed that *ca.* 20% of the total amount of the DOX was loaded physically inside the NGs.

### ^1^H NMR characterization

Proton nuclear magnetic resonance (^1^H NMR) spectra were recorded using an Avance III 400 MHz spectrometer (Bruker, Switzerland). The samples were dissolved in deuterated dimethyl sulfoxide or D_2_O.

### TEM observation

TEM images of Dex-SS-PAA NGs were loaded onto a carbon support, and stained using a 2 wt.% phosphotungstic acid solution for 30 min before being recorded using a JEM-2100 (JEOL, Japan, 200 kV) instrument. Meanwhile, DOX-loaded NGs were loaded onto ultra-thin carbon films and measured using a Tecnai G2 spirit Biotwin BIO-TEM (FEI Co., 120 kV).

### Light scattering

Hydrodynamic diameter and Zeta potential measurements were performed using a Zetasizer Nano ZS90 instrument (Malvern, U.K.) at a concentration of 1 mg/mL and at room temperature. Static light scattering (SLS) experiments were performed using an ALV/CGS-8F S/N 064 laser goniometer system equipped with an ALV/LSE-5004 Multiple Tau Digital Correlator and a He-Ne laser (λ_0_ = 632.8 nm). Prior to the SLS measurements, the sample solutions were filtered using Millipore filters with a pore diameter of 0.45 μm and all of the SLS measurements were performed at 25 °C, with a scattering angle range of 30°−150°. The d*n*/d*c* values were measured using a Kejian Laser Differential Refractometer.

### Redox-responsiveness of the Dex-SS-PAA NGs

To evaluate the redox sensitivity of the Dex-SS-PAA NGs, a reduced L-Glutathione (GSH) solution (Control experiments were also performed with the addition of an equal volume of pure water) was added into a 1 mg/mL aqueous solution of the NGs to reach a final concentration of 10 mM under nitrogen protection and with gentle stirring. The sample was collected at different time intervals, the sizes and the light scattering intensities of the sample were monitored with a Zetasizer Nano ZS90 instrument. The entire process lasted for 3 h. The *M*_w_ values of the samples were obtained through SLS measurements. TEM image of Dex-SS-PAA at 30 min after adding GSH was loaded onto a carbon support and measured using a Tecnai G2 spirit Biotwin BIO-TEM (FEI Co., 120 kV).

### DOX content determination and *in vitro* drug release

The DOX content was determined for the 1 mg/mL DOX-loaded solution using a Shimadzu UV–2550 UV-Vis spectrophotometer. Various concentrations of DOX solutions were used to calibrate the absorption of Dex-SS-PAA-DOX NCs at 480 nm. The drug loading content (LC) and drug encapsulation efficiency (EE) were calculated according to Formula 1 and 2:^45^


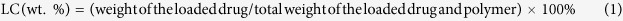



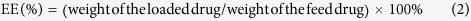


The *in vitro* DOX release test was conducted *via* the dialysis method. In summary, 1 mL of a 5 mg/mL solution was transferred into a dialysis bag (molecular weight cutt-off = 3,500 Da) and immersed into 9 mL of phosphate buffer saline solutions (PBS, pH = 7.4, 0.01 M, addition 0 or 10 mM GSH), or acetate buffer saline solutions (pH = 5.0, 0.01 M, addition 0, 1 or 10 mM GSH), and magnetically stirred at 37 °C with a speed of 120 rpm. In order to measure the DOX release profiles, all of the surrounding solutions were withdrawn and replaced with equal volumes of corresponding fresh buffer solutions at various time points.

### Cell viability assays

Cell viability assay measurements of the samples were carried out on Human Umbilical Vein Endothelial Cells (HUVEC), MCF-7, MDA-MB-231 cells with MTT assays. The cells were purchased from the Shanghai Institute of Cell Biology, the Chinese Academy of Sciences (Shanghai, China). Cells were seeded at a density of 2000 cells/well in 96-well cell culture plates, and incubated under 5% CO_2_ at 37 °C for 72 h with NGs. Various equivalent DOX concentrations (0, 2.5, 5, 10, 15, 20, 25, 30, 35 μg/mL) were added into each well (n = 5).

### Cellular uptake observation by fluorescence microscopy

Cells were seeded in 6-well cell culture plates, and subsequently incubated for 12 or 48 h in DMEM medium. Samples were added into the medium to reach a final DOX concentration of 15 μg/mL. After co-incubation, the cells were washed three times with PBS, fixed with 4 wt.% paraformaldehyde for 25 min and the cellular nuclei were stained with DAPI before they were observed under a fluorescence microscope (IX-70, Olympus, Japan).

### *In vivo* tumor ablation study

An *in vivo* study was performed on 4–5 week old nude mice (Animal Institute of Chinese Academy of Sciences, Shanghai, China). All procedures were approved by the Shanghai Jiao Tong University Animal Care and Use Committee and all the animals were kept in standardized conditions under Chinese NIH guidelines for the care and use of laboratory research animals. All experiments were performed in accordance with relevant guidelines and regulations. To establish human breast cancer xenografts, a cellular suspension (200 μL) containing 5 × 10^6^ MCF-7 cells in normal saline was injected subcutaneously into axillary subcutaneous tissue the frank region of the mice. When the tumor had reached approximately 50–150 mm^3^ in volume, the mice were randomly assigned into four treatment groups which included three medicated groups and one control group (n = 5). The medicated groups were respectively treated with free DOX, Dex-SS-PAA-DOX NGs and Dex-MBA-PAA-DOX NGs at a DOX dosage of 4 mg/kg every two or three days through the caudal vein. In contrast, the control group was injected with PBS. The treatment lasted for 26 days. The weight and tumor volume of each mouse was recorded. The tumor volume was calculated according to Formula 3 (the volume of a prolate spheroid^47^):


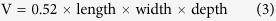


The tumors were weighed and the inhibition ratio (IR) was calculated based on Formula 4:^45^


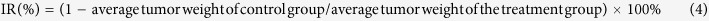


H&E staining and TUNEL staining experiments were performed following the manufacturer’s instructions to evaluate histological change and cell apoptosis of the MDA-MB-231 tumor. The livers, kidneys, spleens, and hearts of the mice treated were also stained with H&E to evaluate the safety of our NGs.

## Additional Information

**How to cite this article**: Wang, H. *et al*. Self-Assembly Assisted Fabrication of Dextran-Based Nanohydrogels with Reduction-Cleavable Junctions for Applications as Efficient Drug Delivery Systems. *Sci. Rep.* **7**, 40011; doi: 10.1038/srep40011 (2017).

**Publisher's note:** Springer Nature remains neutral with regard to jurisdictional claims in published maps and institutional affiliations.

## Supplementary Material

Supplementary Information

## Figures and Tables

**Figure 1:**
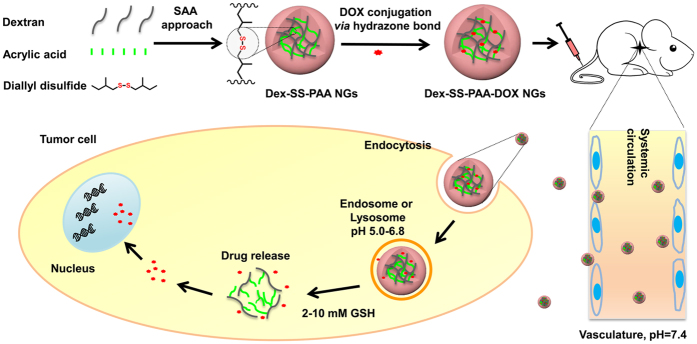
Schematic illustration depicting the fabrication of the Dex-SS-PAA NGs, as well as their subsequent loading with DOX and their tumor-microenvironment sensitive drug release behaviors.
