# Supplementary material for: Self-Assembly Assisted Fabrication of Dextran-Based Nanohydrogels with Reduction-Cleavable Junctions for Applications as Efficient Drug Delivery Systems
Source: Sci Rep. 2017 Jan 10;7:40011. doi: 10.1038/srep40011 (PMC5223173; doi:10.1038/srep40011)
Supplement: Supplementary Information [file srep40011-s1.pdf]

**Supplementary information**

# Self-Assembly Assisted Fabrication of Dextran-Based Nanohydrogels with Reduction-Cleavable Junctions for Applications as Efficient Drug Delivery Systems

Hao Wang[1, †], Tingting Dai[2, †], Shuyan Zhou[1], Xiaoxiao Huang[1], Songying Li[1], Kang Sun[1], Guangdong Zhou[2], Hongjing Dou[1]

[1]The State Key Laboratory of Metal Matrix Composites, School of Materials Science and Engineering, Shanghai Jiao Tong University, Shanghai 200240 (P. R. China).

[2]Department of Plastic and Reconstructive Surgery, Shanghai ninth People's Hospital, Shanghai Jiao Tong University School of Medicine, Shanghai Key Laboratory of Tissue Engineering, National Tissue Engineering Center of China, Shanghai 200011 (P. R. China).

[†]These authors contributed equally to this work. Correspondence and requests for materials should be addressed to H.D. (hjdou@sjtu.edu.cn) or G.Z. (guangdongzhou@126.com).

**Supplementary information**

**Materials.** Dextran ($M_w$ = 40,000 Da), ceric ammonium nitrate (CAN), methacrylic acid (MAA) and acrylic acid (AA) were purchased from Shanghai Sinopharm Chemical Reagent Co., Ltd. MAA and AA were initially dried over $MgSO_4$ and then distilled under reduced pressure prior to use. CAN was recrystallized from a 3.7 M nitric acid solution containing 11.4 M of ammonium nitrate. Diallyl disulfide (DADS, 80%) was purchased from Sigma Aldrich and used after the removal of impurities (mainly allyl mercaptan) by vacuum distillation. *N,N'*-Methylene bisacrylamide (MBA, Fluka) was recrystallized from methanol. Doxorubicin hydrochloride (DOX, 99%) was purchased from Beijing ZhongShuo Pharmaceutical Technology Development Co., Ltd. L-Glutathione reduced (GSH, ≥98.0%), 4',6-diamidino-2-phenylindole dihydrochloride (DAPI) and 3-(4,5-dimethylthiazol-2-yl)-2,5-diphenyl tetrazolium bromide (MTT) were purchased from Sigma-Aldrich. Dulbecco's modified eagle medium (DMEM) and fetal bovine serum (FBS) were purchased from Hyclone, while penicillin and streptomycin were purchased from Gibco. All other chemical reagents (DMSO, $MgSO_4$, $D_2O$ and deuterated DMSO) were purchased from Shanghai Sinopharm Chemical Reagent Co., Ltd. DMSO was dried over $CaH_2$ and subsequently distilled under reduced pressure before use. Dialysis membrane bags with a cut-off molecular weight of 14,000 Da were purchased from Shanghai Green Bird Co., Ltd.

## Supplementary information

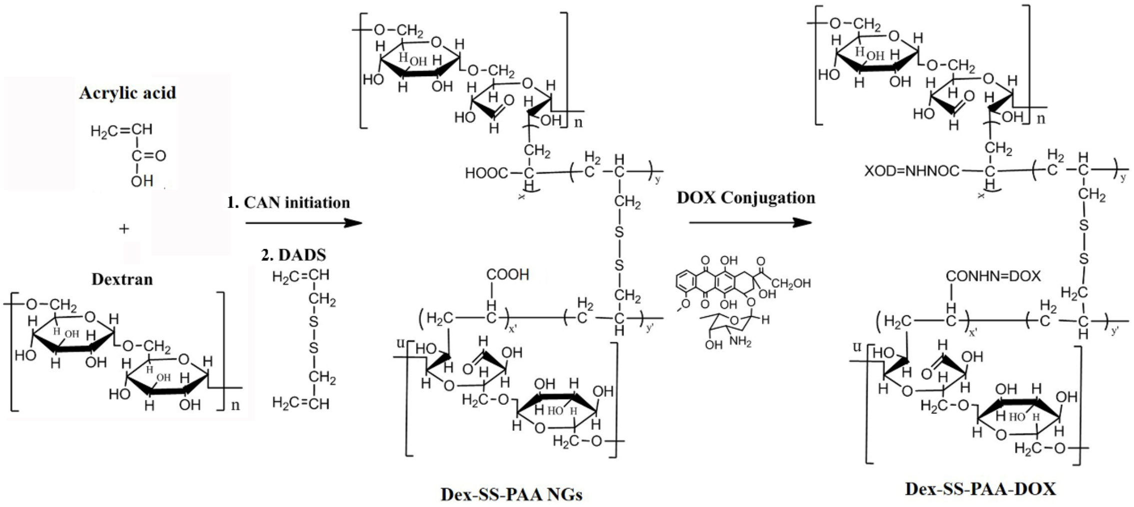


**Figure S1.** Synthesis of the Dex-SS-PAA and the Dex-SS-PAA-DOX NGs.

**Supplementary information**

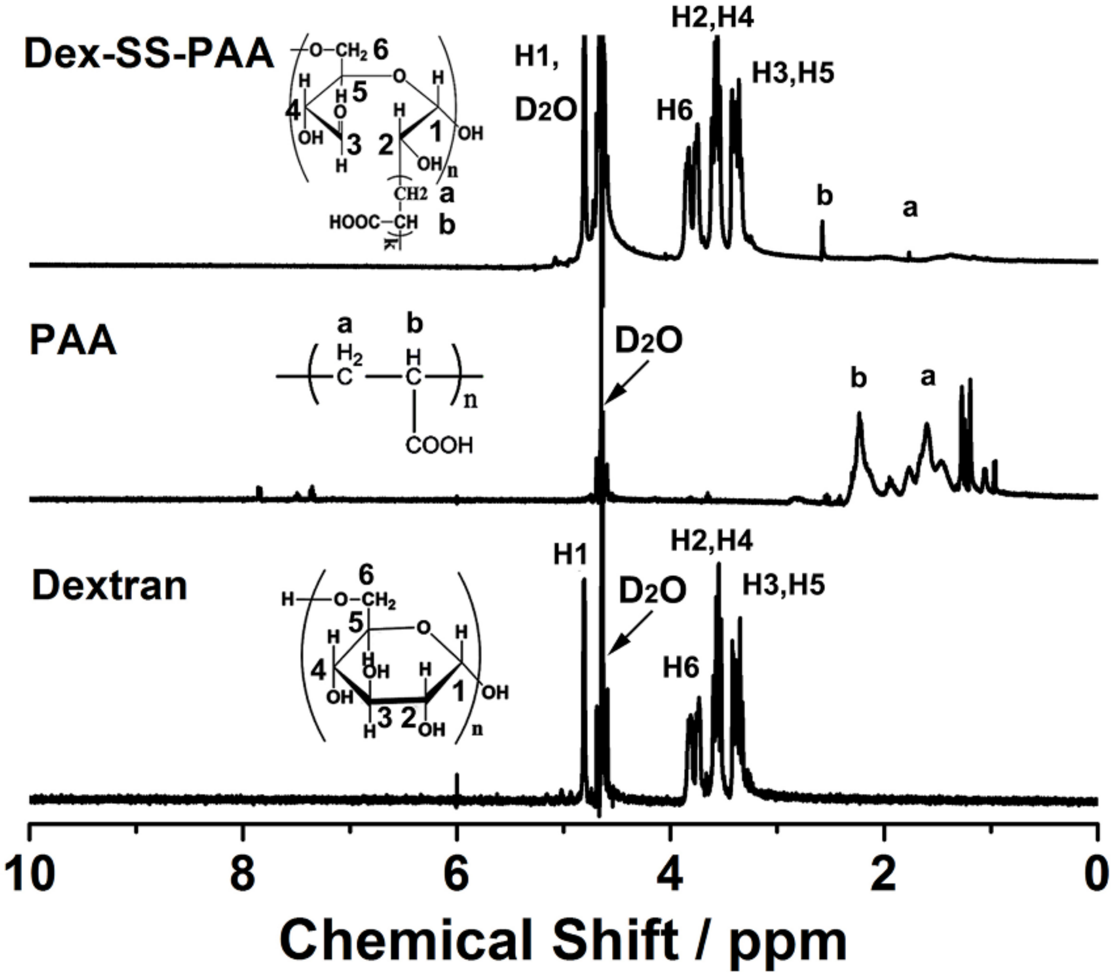


**Figure S2.** $^{1}$H NMR spectra ($D_2O$) of dextran, PAA and Dex-SS-PAA NGs.

**Supplementary information**

**Table S1.** DLS characterization details of Dex-SS-PAA NGs prepared at different AA-to-dextran unit molar ratios.

| Sample | $n_{Monomer}$: $n_{Glu}$ [a] | $n_{Glu}$: $n_{Initiator}$ | $n_{Monomer}$: $n_{Crosslinker}$ | $\langle D_h \rangle$ [nm] | PDI | Zeta Potential / mV |
|---|---|---|---|---|---|---|
| Dex-SS-PAA (S) | 0.5:1 | 7:1 | 10:1 | 37.5 ± 0.2 | 0.312 | -4.9 ± 1.5 |
| Dex-SS-PAA | 1:1 | 7:1 | 10:1 | 98.0 ± 5.0 | 0.191 | -10.0 ± 0.7 |
| Dex-SS-PAA (L) | 2:1 | 7:1 | 10:1 | 944.3 ± 66.5 | 0.805 | -7.4 ± 0.3 |

a) $n$ is the molar amount, $n_{Glu}=[m_{Dex}/M_{wDex}]/M_{wGlu}$, where $M_{wGlu}$=168, $m_{Dex}$ and $M_{wDex}$ are the weight and weight-average molecular weight.

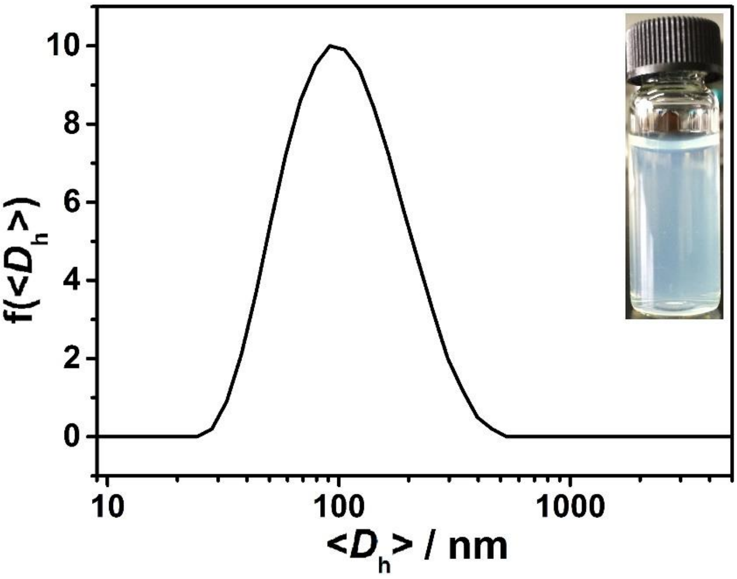


**Figure S3.** The size distribution of Dex-SS-PMAA NGs (<$D_h$> = 90.6 nm, PDI = 0.255, zeta potential = -9.5 mV), the inset shows a photograph of the Dex-SS-PMAA NGs aqueous solution at a concentration of 29 g/L.

**Supplementary information**

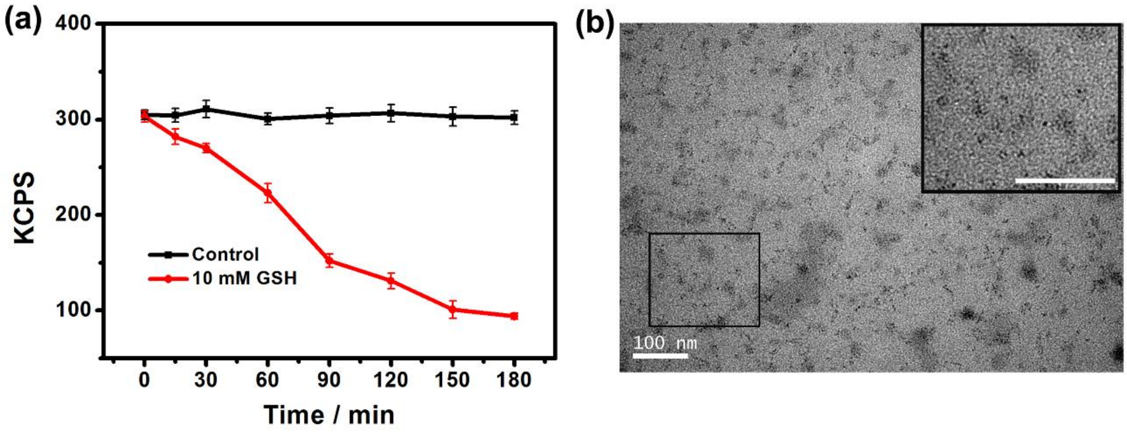


**Figure S4.** (a) Plots of the kilo counts per second (KCPS) of the Dex-SS-PAA NGs solution after the addition of 10 mM GSH as functions of time. (b) TEM image of Dex-SS-PAA at 30 min after adding GSH. Scale bar: 100 nm in the inset.

**Supplementary information**

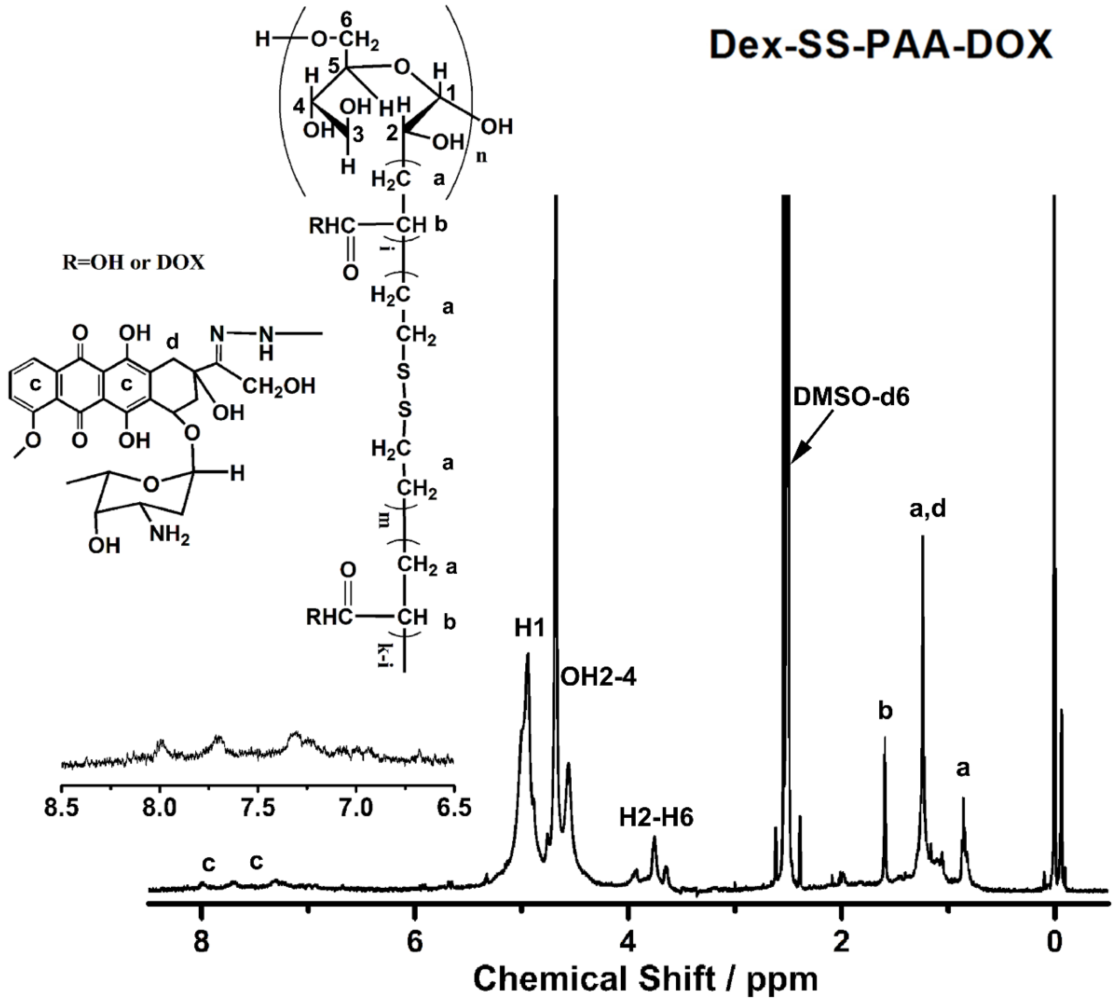


**Figure S5.** $^{1}$H NMR spectrum of Dex-SS-PAA-DOX NGs.

**Supplementary information**

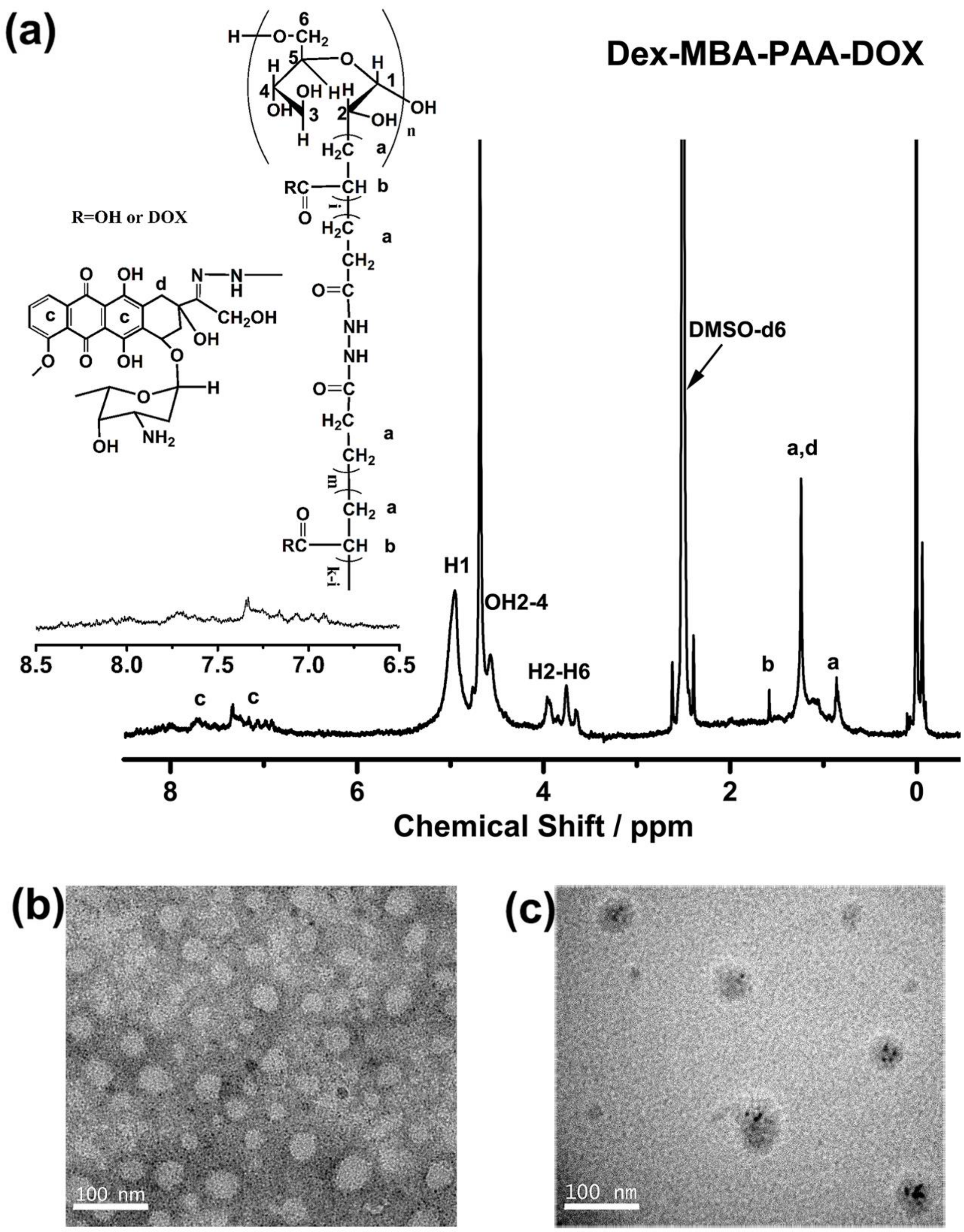


**Figure S6.** (a) $^1$H NMR spectrum of Dex-MBA-PAA-DOX. TEM images of (b) Dex-MBA-PAA and of (c) Dex-MBA-PAA-DOX.

## Supplementary information

**Table S2.** DLS measurements of Dex-MBA-PAA NGs and Dex-MBA-PAA-DOX.

| Sample | <$D_h$> / nm | PDI | Zeta Potential / mV |
|---|---|---|---|
| Dex-MBA-PAA NGs | 107.2 ± 2.6 | 0.139 | -10.1 ± 1.0 |
| Dex-MBA-PAA-DOX | 116.4 ± 9.8 | 0.265 | -33.2 ± 2.5 |

**Supplementary information**

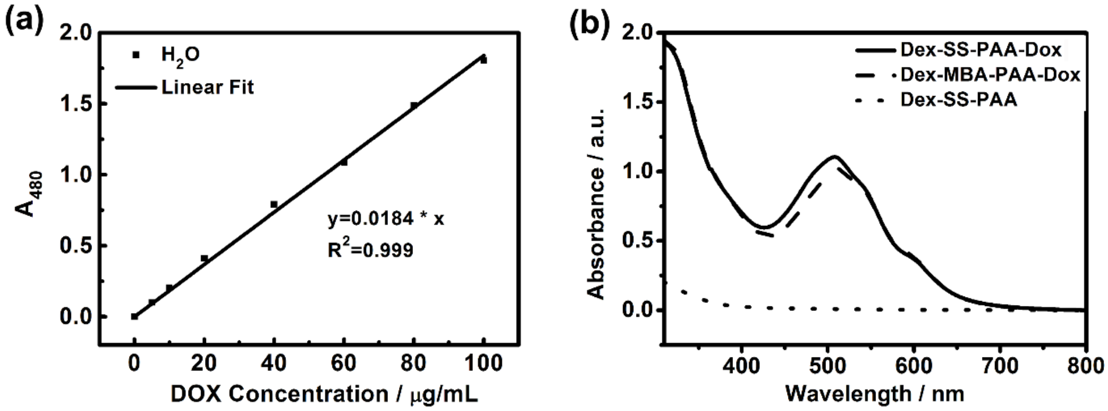


**Figure S7.** (a) The UV-Vis absorbance calibration curve of various concentrations of DOX solutions at 480 nm. (b) The UV-Vis absorbance spectra of Dex-SS-PAA NGs, Dex-SS-PAA-DOX NGs and Dex-MBA-PAA-DOX NGs (1 mg/mL).

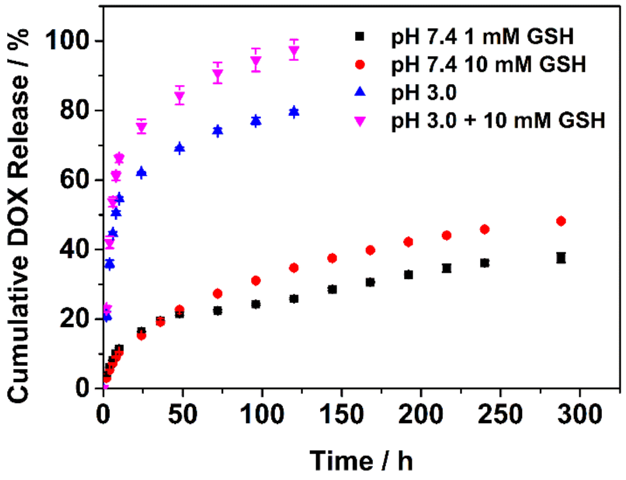


**Figure S8.** *In vitro* drug release profiles of Dex-SS-PAA-DOX NGs at various GSH concentrations.

**Supplementary information**

**Table S3.** pH values of different types of PBS buffers with GSH addition

| pH and concentration of phosphate buffer (before GSH addition) | pH 1 mM GSH addition | pH 5 mM GSH addition | pH 10 mM GSH addition |
|---|---|---|---|
| 7.4 (0.01 M) | 7.1 | 6.5 | 5.0 |
| 5.0 (0.01 M) | 5.0 | 4.3 | 3.8 |
| 7.4 (0.05 M) | 7.2 | 7.0 | 6.7 |
| 5.0 (0.05 M) | 5.0 | 4.9 | 4.7 |

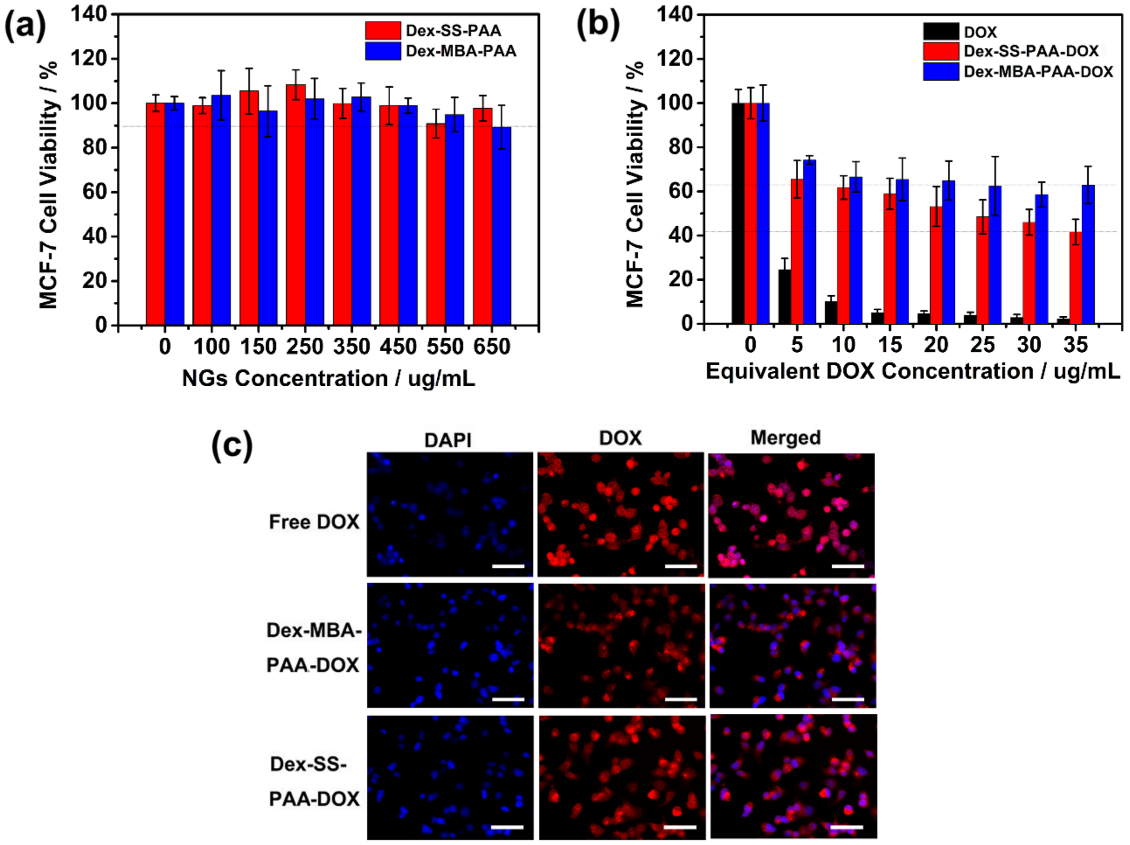


**Figure S9.** (a) MCF-7 cell viability of non-drug-loaded Dex-SS-PAA NGs and Dex-MBA-PAA NGs. (b) MCF-7 cell inhibition capabilities of free DOX, Dex-SS-PAA-DOX NGs and Dex-MBA-PAA-DOX NGs. (c) Fluorescence microscope images of MCF-7 cells after co-incubation with free DOX, Dex-MBA-PAA-DOX NGs and Dex-SS-PAA-DOX NGs for 48 h at an equivalent DOX concentration of 15 μg/mL. The red colour corresponds to the fluorescence of DOX, while the blue colour corresponds to the fluorescence of DAPI (a cell nucleus dye). Scale bars: 100 μm.
